# Desert Ants Learn Vibration and Magnetic Landmarks

**DOI:** 10.1371/journal.pone.0033117

**Published:** 2012-03-07

**Authors:** Cornelia Buehlmann, Bill S. Hansson, Markus Knaden

**Affiliations:** Max Planck Institute for Chemical Ecology, Jena, Germany; Arizona State University, United States of America

## Abstract

The desert ants *Cataglyphis* navigate not only by path integration but also by using visual and olfactory landmarks to pinpoint the nest entrance. Here we show that *Cataglyphis noda* can additionally use magnetic and vibrational landmarks as nest-defining cues. The magnetic field may typically provide directional rather than positional information, and vibrational signals so far have been shown to be involved in social behavior. Thus it remains questionable if magnetic and vibration landmarks are usually provided by the ants' habitat as nest-defining cues. However, our results point to the flexibility of the ants' navigational system, which even makes use of cues that are probably most often sensed in a different context.

## Introduction

Ants are equipped with sophisticated navigational skills (for reviews see [1], [2], [3], [4]). Multiple orientation cues are available in the ants' environment that can be used to return to the nest. The individually foraging desert ants of the genus *Cataglyphis* perform path integration during foraging that takes into account the ants' walking distances and directions and continuously provides the ants with a home vector that points back to the nest entrance, a tiny hole in the desert ground [5], [6]. Compass information (mainly based on polarized skylight [7], but also on the position of the sun and even on wind direction [8]) provides the ants with directional information while a step integrator informs them about the distances covered [9]. Since path integration is error prone [10], [11], *Cataglyphis* ants also use visual [12], [13], olfactory [14], [15], [16] and tactile landmarks [17] to pinpoint their nest. In studies with other ant species gravity [18] and the earth's magnetic field [19], [20] have been reported to provide directional information. Thermal radiation has been shown to be perceived and used in leaf-cutting ants for relocation of brood and fungus [21], [22] while vibrational signals are used for communication about food sources or buried nest mates [23], [24].

Taken together, ants have access to a large variety of potential cues. In the present account we provide evidence that *Cataglyphis* ants use this diversity of information sources for navigation. Although probably neither magnetic nor vibrational landmarks are provided by the ants' habitat as nest-defining cues, *C. noda* foragers were able to associate a magnetic landmark and a local vibration with the nest entrance.

## Results and Discussion

**Figure 1:**
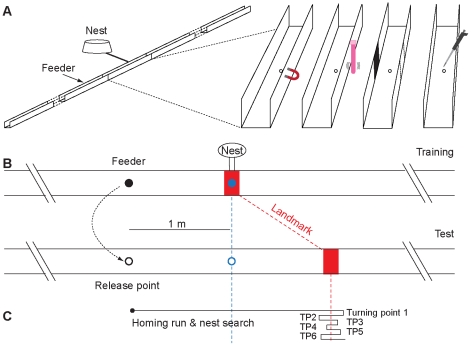
Experimental procedure. (A) The ants' nest was connected with a tube to the training channel where the ants were trained to visit a feeder 1 m away from the nest entrance that was marked with either a magnetic, vibrational, visual, olfactory or no landmark. For size and shape of the solenoid, and for the application of the massaging rod next to the channel see Material and Methods. (B) Trained ants were displaced from the feeder of the training channel into the parallel test channel (displacement shown by dashed arrow) where the homing runs and nest searches of the tested ants were tracked and recorded. Blue filled circle, nest entrance; black filled circle, feeder; black empty circle, release point; blue empty circle, fictive nest position, red rectangle, landmark; blue dashed line, nest position as defined by path integration, red dashed line, nest position as defined by landmark. Nest-to-feeder distance, 1 m; landmark was 1 m behind fictive nest position in test channel. (C) Exemplar homing run and nest search. We analyzed the first six turning points (TP1–TP6) after the ants had crossed the nest-defining cue for the first time.

**Figure 2:**
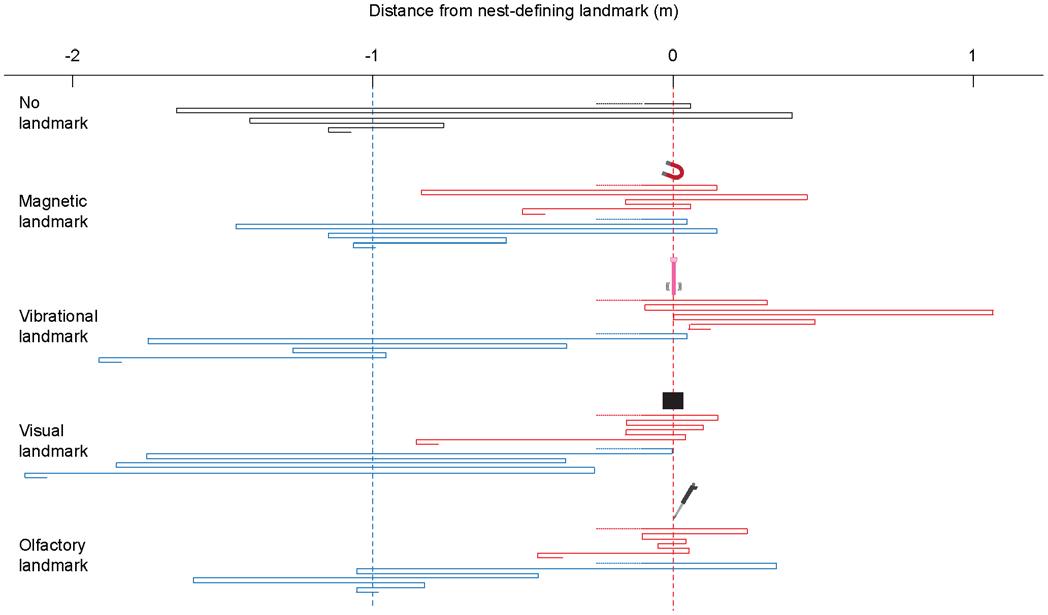
Individual test runs of homing ants. Schematic nest searches of ants trained and tested with a nest-defining landmark that was either a magnetic, vibrational, visual or olfactory cue (red), control ants trained and tested without landmark (black) or naïve ants that experienced the landmark in the test for the first time (blue). Blue dashed line, nest position as defined by path integration; red dashed line, nest position as defined by landmark; point of release for each homing run at position -2 m from nest-defining cue. The first six turning points after the ants had passed the landmark for the first time were analyzed for their median position.

**Figure 3:**
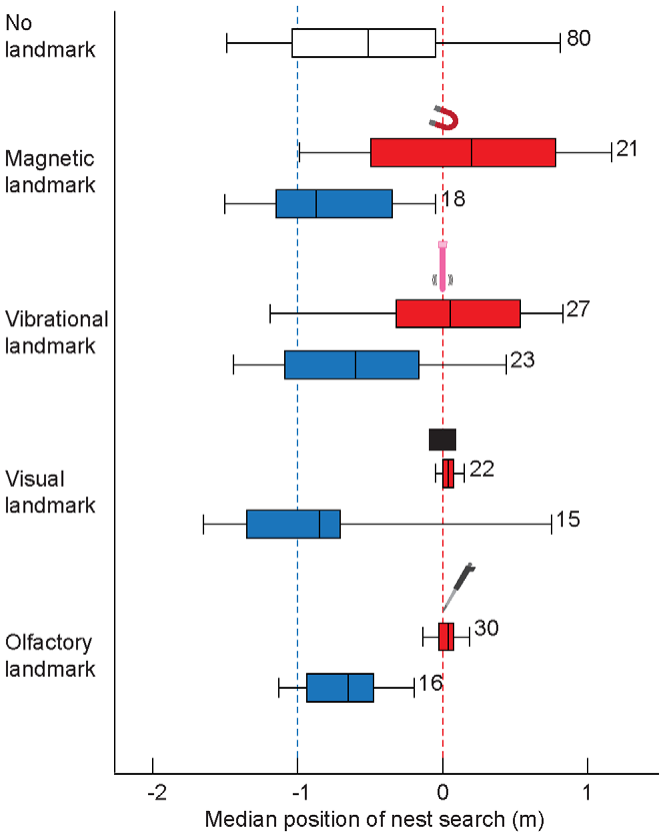
Ants learn magnetic, vibrational, visual and olfactory landmarks. Box plot representation of the medians of the first six turning points of ants that were trained and tested with a landmark (red boxes), control ants trained and tested without a landmark (white box), and naïve ants that experienced the landmark during the test for the first time (blue boxes). Blue dashed line, nest position as defined by path integration; red dashed line, nest position as defined by landmark. Box plots show median, interquartile range and whiskers indicating the 90th and 10th percentiles. Kruskal-Wallis test and Dunn's multiple comparison tests were performed for selected pairs: control (training and test without landmark) versus naïve ants (landmark only during test), for each landmark type P>0.05; ants trained and tested with the landmark versus naïve ants, for each landmark type P<0.05; ants trained and tested with the landmark versus control ants, for each landmark type P<0.05. Numbers depict sample sizes.

We trained and tested ants in a channel with either a magnetic, vibrational, visual, or olfactory nest-defining landmark (Figure 1) and compared the nest-search performances of these ants with those of ants that either were trained and tested without landmark (control ants) or naïve ants that experienced the landmark in the test situation for the first time. To investigate whether the ants relied on landmarks or on path-integration, we established a conflict between these two sources of information (see Figure 1B and also Material and Methods). Control ants (training and test without landmark) searched near the nest position defined by the path integrator (Figures 2 and 3). The same was true for naïve ants that experienced the landmark in the test channel for the first time (Figures 2 and 3), indicating that the landmarks were not innately attractive to the ants. However, ants that were trained with a landmark as a nest-defining cue and later tested with this cue focused their search at the landmark (Figures 2 and 3). Hence, our results suggest that *C. noda* foragers were able to learn and use all provided cues – be they magnetic, vibrational, visual or olfactory information – in order to locate the nest position.

It is well known that ants orientate by using visual and olfactory cues (see above), but debate continues on whether and how they use the magnetic sense for orientation. In studies dealing with the magnetic sense of ants a change of the magnetic field's polarity resulted in disturbed homing behavior [19], [20]. However, navigation by using the magnetic field as a compass does not seem to be the primary mechanism in ant navigation [25]. Our data suggest that apart from using magnetic cues for compass information *Cataglyphis* ants can learn and use a magnetic landmark as a nest-defining landmark. The use of positional information derived from local anomalies of the earth's magnetic field has been shown also for other animals, e.g. for sea turtles, birds, and spiny lobsters [26], [27]. Furthermore bees can be trained to visit a feeder that is equipped with a changed magnetic field [28]. However, it remains questionable whether any natural magnetic anomalies exist that on a scale of a few meters could help ants to localize their nest entrance. Furthermore our finding does not necessarily suggest any specialized magnetic-sensitive organ in *Cataglyphis*, as the strong change of the magnetic field induced by the solenoids (see Material and Methods) could potentially have led to an unspecific change of neuronal activity that later was associated with the nest entrance. While the existence of a magnetic sense in ants is still under discussion, the use of vibrational signals is well investigated. Ants are very sensitive to vibration [23], [24]. However, our finding that *Cataglyphis* learns vibrational nest-defining landmarks is surprising. Buried leaf-cutting ants call for help via vibrations that are sensed by nest mates through several centimeters of nest material [24]; hence, it is within the realm of possibility that *Cataglyphis* foragers can sense the whole community below the nest entrance. However, it remains an open question if vibrational landmarks exist and are used by these ants in their natural habitat as nest-defining cues.

Our findings highlight the flexibility of the ants' navigation system. Not only can they associate visual and olfactory cues with the nest entrance, but they can apparently also learn magnetic and vibrational cues that are typically sensed in a completely different context.

## Materials and Methods

### Field Site and Ant Species

The experiments with the desert ants *Cataglyphis noda* (Brullé, 1832) were performed between mid June and July 2011 in the ants' natural habitat. The field site was located in Çirali, Turkey (36°25′N, 30°29′E). No specific permits were required for the described field studies.

### Experimental Procedure

#### Training Procedure

The ants' nest was covered with a bucket and connected to a U-shaped linear channel (cross section, total length: 19.5 m, width: 7 cm, height: 7 cm) so that the ants could enter the training channel by a tiny hole in the channel floor (Figure 1A). We trained *C. noda* foragers in this channel to visit a feeder 1 m upwind of the nest entrance that was marked with one of the following nest-defining cues (Figure 1A).

Magnetic landmark: As a magnetic landmark we used two solenoids (circular nickel-coated neodymium magnets, 5 mm in diameter and 10 mm high, volume: 393 mm^3^, NdFeB magnet in N45) placed adjacent to the nest entrance on the outer walls of the aluminum channel. They caused a 180° reversal in the polarity and an increase in the intensity of the magnetic field (maximal intensity of 21000000 nT measured within the channel, compared to the earth's magnetic field strength of 41000 nT).

Vibrational landmark: We put a massaging rod into the ground outside of the channel next to the position of the nest entrance. Apart from the vibrations within the channel we could not measure any changes in the magnetic field in the presence of the rod. In order to exclude that the ants learned minor magnetic effects rather than vibrational effects of the rod, we in addition trained and tested ants in the presence of a vibrating rod that was placed close to the nest but had no contact to the ground (i.e. did not generate vibrations). The nest-search performances of the tested ants did not differ from those of control ants that were trained and tested without the rod (Mann-Whitney test, P>0.05, data not shown).

Visual landmark: Two pieces of black cardboard (each 10 cm×7 cm) that were placed adjacent to the nest entrance on the inner walls of the channel were used as a visual landmark.

Olfactory landmark: As an olfactory landmark we dropped dilute methyl salicylate (1∶50 in hexane) directly at the nest entrance on the channel floor (see also [14]). Due to evaporation we renewed the olfactory landmark every 15 min.

#### Test Procedure

Trained ants were captured at the feeder and together with a food crumb were released into an aligned test channel so that they were still equipped with the path-integration vector that guided them to the fictive nest position (Figure 1B). The nest-defining cue that was presented in the training channel was placed 1 m behind the nest position as defined by path integration. The conflict between path integration and landmark information allowed us to investigate whether the ants were relying on landmark information or on path-integration information. When an ant does not reach the nest entrance after it has run off its path-integration vector it starts a systematic nest search [29], [30]. Within the linear test channel this systematic nest search is reduced to one dimension and is characterized by the turning points [31]. We tracked and recorded the turning points (TP) of the homing ants by aligning a measuring tape along the channel outer wall.

### Analyses and Statistics

The first six turning points after the ants had crossed the nest-defining cue for the first time were analyzed for their median position (Figure 1C). Analyses had to be restricted to the first six turning points, because many tested ants managed to leave the channel afterwards. The non-parametric Kruskal-Wallis test with Dunn's multiple comparison tests and the Mann-Whitney test were performed throughout the analyses with the statistic software GraphPad Instat (version 3.06). We only analyzed ants that took a food crumb and crossed the landmark position within the first 11 turning points.
